# Targeted Oral Delivery of Paclitaxel Using Colostrum-Derived Exosomes

**DOI:** 10.3390/cancers13153700

**Published:** 2021-07-23

**Authors:** Raghuram Kandimalla, Farrukh Aqil, Sara S. Alhakeem, Jeyaprakash Jeyabalan, Neha Tyagi, Ashish Agrawal, Jun Yan, Wendy Spencer, Subbarao Bondada, Ramesh C. Gupta

**Affiliations:** 1 James Graham Brown Cancer Center, University of Louisville, Louisville, KY 40202, USA; raghuram.kandimalla@louisville.edu (R.K.); farrukh.aqil@louisville.edu (F.A.); neha.tyagi@louisville.edu (N.T.); ashish.phe@iitbhu.ac.in (A.A.); jun.yan@louisville.edu (J.Y.); 2 Department of Pharmacology and Toxicology, University of Louisville, Louisville, KY 40202, USA; 3 Department of Medicine, University of Louisville, Louisville, KY 40202, USA; 4 Department of Microbiology, Immunology & Molecular Genetics, University of Kentucky, Lexington, KY 40536, USA; sara.alhakeem@bioagilytix.com (S.S.A.); subbarao.bondada@uky.edu (S.B.); 5 3P Biotechnologies, Inc., Louisville, KY 40202, USA; jp3pbiotech@gmail.com (J.J.); wendyspencer3p@gmail.com (W.S.); 6 Department of Surgery, University of Louisville, Louisville, KY 40202, USA

**Keywords:** colostrum exosomes, paclitaxel, drug delivery, lung cancer, immunotoxicity assessment

## Abstract

**Simple Summary:**

Paclitaxel (PAC) is a widely used antitumor agent in the treatment of various early-stage and advanced cancers, including lung cancer. While efficacious, solvent-based PAC generally is not well tolerated and is associated with severe side effects. To overcome such limitations, naturally occurring nanocarriers such as exosomes are attracting great interest. In this paper, we show that tumor-targeted oral formulation of PAC, using bovine colostrum-derived exosomes, not only enhance therapeutic efficacy against orthotopic lung cancer but also mitigate or eliminate systemic and immunotoxicity of the conventional i.v. dosing. These data will leverage the advantages of bovine colostrum exosomes to advance the exosome-mediated targeted oral delivery of PAC as a therapeutic alternative to current therapies.

**Abstract:**

Lung cancer is the leading cause of cancer-related deaths worldwide. Non-small-cell lung cancer (NSCLC) is the most common type accounting for 84% of all lung cancers. Paclitaxel (PAC) is a widely used drug in the treatment of a broad spectrum of human cancers, including lung. While efficacious, PAC generally is not well tolerated and its limitations include low aqueous solubility, and significant toxicity. To overcome the dose-related toxicity of solvent-based PAC, we utilized bovine colostrum-derived exosomes as a delivery vehicle for PAC for the treatment of lung cancer. Colostrum provided higher yield of exosomes and could be loaded with higher amount of PAC compared to mature milk. Exosomal formulation of PAC (ExoPAC) showed higher antiproliferative activity and inhibition of colony formation against A549 cells compared with PAC alone, and also showed antiproliferative activity against a drug-resistant variant of A549. To further enhance its efficacy, exosomes were attached with a tumor-targeting ligand, folic acid (FA). FA-ExoPAC given orally showed significant inhibition (>50%) of subcutaneous tumor xenograft while similar doses of PAC showed insignificant inhibition. In the orthotopic lung cancer model, oral dosing of FA-ExoPAC achieved greater efficacy (55% growth inhibition) than traditional i.v. PAC (24–32% growth inhibition) and similar efficacy as i.v. Abraxane (59% growth inhibition). The FA-ExoPAC given i.v. exceeded the therapeutic efficacy of Abraxane (76% growth inhibition). Finally, wild-type animals treated with *p.o.* ExoPAC did not show gross, systemic or immunotoxicity. Solvent-based PAC caused immunotoxicity which was either reduced or completely mitigated by its exosomal formulations. These studies show that a tumor-targeted oral formulation of PAC (FA-ExoPAC) significantly improved the overall efficacy and safety profile while providing a user-friendly, cost-effective alternative to bolus i.v. PAC and i.v. Abraxane.

## 1. Introduction

Cancer is the second leading cause of death worldwide. In 2020, there were an estimated 1.8 million new cancer cases diagnosed and 606,520 cancer deaths in the United States [1]. More people in the U.S. (135,760) are expected to die of lung cancer in 2021 than prostate, breast and colon cancer combined [2]. Lung cancer remains the leading cause of cancer-related deaths in the United States and worldwide. Non-small cell-lung cancer (NSCLC) is relatively insensitive to chemotherapy and accounts for about 85% of all lung cancer cases. Regrettably, over 80% of all patients diagnosed with NSCLC die eventually due to the disease within five years [1,3]. Despite treatment with platinum-based chemotherapy, new molecularly-targeted therapies and immunotherapies, the overall survival benefit for NSCLC remains modest.

Paclitaxel (PAC) is the first- or second-line chemotherapy for the treatment of various cancers, including lung cancer and exhibits both anti-proliferative and apoptotic effects against cancer cells. Mechanistically, PAC interferes with the normal function of cellular microtubule growth by binding to the β-subunits of the tubulin and locking the microtubules preventing further cell division. Tubulins are the building blocks of microtubules, which play a major role in the migration of chromosomes during anaphase of the cell division [4]. However, the utility and clinical application of PAC has been hindered due to its poor aqueous solubility requiring formulation in the organic solvent Cremophor EL (CrEL) and its dose-related toxicity. For these reasons, the delivery of PAC is associated with substantial challenges. While the use of polyoxyethylated castor oil also known as CrEL and ethanol (50:50) overcomes the solubility problem, this solvent-based approach is associated with severe side effects [5,6]; therefore, PAC formulations are infused over several hours to reduce the effect of bolus dose.

To overcome these solvent-based limitations, several nanoparticle systems have been reported for the delivery of PAC. Abraxane^®^ is an FDA-approved nanoformulation of PAC bound to human serum albumin that was developed to improve the toxicity profile of solvent-based PAC. In a phase III clinical trial, Abraxane was shown to enhance the therapeutic efficacy and pharmacokinetics compared to PAC given in CrEL [7]. However, the i.v. infusion of the Abraxane was reported to lower the blood cell count. Besides toxicity concerns, i.v. administration requires medical assistance, which, in turn, substantially increases the medical costs, besides patient suffering for a long duration.

To overcome these unfavorable physicochemical and pharmacokinetic properties of PAC, several additional delivery approaches have been attempted [8]. Toxicity limitations of solvent-based carriers can be overcome by using nanovesicles derived from natural sources such as milk [9,10]. Further, oral dosing of the chemotherapeutic achieved using these nanovesicles has many advantages such as flexibility of timing and location of administration, flexibility of drug exposure, reduction of the use of healthcare resources and a better quality of life [11,12]. Oral chemotherapy is also good for the metronomic (anti-angiogenic) chemotherapy [13], as it maintains a low serum level of the chemotherapeutic for a longer time than parenteral routes.

Exosomes (Exo) or small extracellular vesicles (sEVs), as the terminology is being debated [14], are biogenic nanocarriers (30–150 nm) with the lipid bilayer and have significant role in cell-to-cell communications. Exosomes are released from essentially all cell types and are present in all bodily fluids like blood, urine, saliva, amniotic fluid, lymphatic fluid and milk etc. [15,16]. Unlike other nanoparticulate systems, exosomes possess special proteins in their membrane surface proteins that may help in the endocytosis, which, in turn, promotes the delivery of tethered content [17,18]. We have previously demonstrated the utility of bovine milk as a source of exosomes for the delivery of small-molecule drugs [19,20,21,22] and siRNA [23,24] and for the oral delivery of PAC to inhibit subcutaneous lung tumor xenografts [19]. Biocompatibility, cost-effectiveness and abundance are some of the hallmarks that make milk exosomes a potentially commercially viable option as a nanodrug carrier.

In this study, we used exosomes isolated from a standardized source of bovine colostrum powder obtained from the early lactation period as a delivery vehicle for PAC (ExoPAC). Colostrum powder provides higher yields of exosomes than mature milk. We have shown an overexpression of FRα and RFC in H1299 and A549 lung cancer cells; the overexpression of the folate receptors was more pronounced in tumor tissue versus normal lung tissue (100-fold overexpression) [24]. Here, exosomes, functionalized with folic acid (FA) to target tumor cells, are embedded with PAC (FA-ExoPAC), and the therapeutic efficacy of the formulation was compared with Abraxane for lung tumors grown in a tumor microenvironment. We show that FA-ExoPAC given orally surpassed efficacy of solvent-based PAC and matched efficacy of Abraxane; whereas, i.v. FA-ExoPAC significantly exceeded the efficacy of Abraxane. ExoPAC formulations lacked gross, systemic and immunotoxicity in wild-type mice.

## 2. Materials and Methods

### 2.1. Chemicals and Reagents

PAC was procured from LC laboratories, Woburn, MA, USA. XenoLight D-Luciferin, potassium salt was purchased from PerkinElmer, (Waltham, MA, USA). BCA Protein Assay Kit was procured from ThermoFisher Scientific (Waltham, MA, USA) and folic acid was purchased from Sigma-Aldrich (St. Louis, MO, USA). All other chemicals were of analytical grade.

### 2.2. Isolation of Exosomes

Exosomes were isolated from colostrum powder (Immunodynamics, Inc., Fennimore, WI, USA). Briefly, colostrum powder was rehydrated in deionized water achieving a final concentration of 5% *w*/*v*, and exosomes were isolated by sequential centrifugations (13,000× *g*, 30 min; 65,000× *g*, 60 min; and 135,000× *g*, 2 h, as described [22], followed by removal of residual non-exosomal protein by ultrafiltration. After completion of ultracentrifugation, the supernatant containing free drug was discarded and ExoPAC pellet was washed with PBS. The exosome pellet was suspended in PBS (pH 7.4) and sterilized using 0.22 µM filter. The yield of exosomes was measured by means of exosomal protein concentration by a standard BCA protein assay kit. The exosome suspension (≤6 mg/mL) was stored at −80 °C.

### 2.3. Exosome Characterization

The particle size, polydispersity index (PDI) and zeta potential of the exosomes were determined by Zetasizer (Malvern Instruments Ltd., Malvern, Worcestershire, UK). Particle numbers per milligram of exosomal protein were measured by nanoparticle tracking analyzer (NonoView, Particle Matrix Inc., Grayslake, IL, USA). Samples were analyzed in triplicates. The size of exosomes was confirmed by atomic force microscopy (AFM) as described [22].

### 2.4. FA-Functionalization of Exosomes for Tumor Targeting

We functionalized exosomes with FA, a known tumor-targeting ligand. To stabilize the interaction of FA with exosomal proteins in vivo, we attached FA covalently by using activated FA. Activated FA was prepared using standard EDC (1-ethyl-3-(-3-dimethyl aminopropyl) carbodiimide hydrochloride) and NHS (N-hydroxysuccinimide esters). Free FA was removed using ultrafiltration. The degree of functionalization was achieved by varying FA concentration, and FA loading was determined by releasing the FA from the formulation in the presence of NaOH, followed by recovery of the exosomes. The FA and exosomal proteins were measured by spectrophotometry and BCA assay, respectively, and percent FA loading was calculated.

### 2.5. Loading of PAC on Exosomes

PAC was loaded onto the exosomes as described by us previously [19], except that exosomes used were derived from colostrum powder, the ratio of exosomes to PAC was reduced and harvesting time of ExoPAC formulation by ultracentrifuge was reduced to achieve higher drug loading. Briefly, PAC (dissolved in ethanol: acetonitrile; 1:1 *v*/*v*) was mixed with exosomes (6 mg/mL in PBS), keeping the solvent concentration ≤10%. The reaction mixture was incubated at room temperature for 30 min. The unbound PAC was removed by centrifugation (10,000× *g* for 10 min) and the exosomal PAC (ExoPAC) was collected by ultracentrifugation (135,000× *g* for 90 min). The resulting pellet was suspended in PBS and filter-sterilized. The ExoPAC solution (≤6 mg/mL) was stored at −80 °C.

### 2.6. Determination of PAC Loading

PAC loading was determined by analyzing the PAC and Exo concentrations using ultra-performance liquid chromatography (UPLC) and BCA protein estimation kit, respectively, as described [19]. Briefly, 50 µL of the ExoPAC formulation was added to 950 µL of acetonitrile to extract the PAC and precipitate the Exo protein. The reaction mixture was then centrifuged (10,000× *g* for 10 min) to separate the pellet. Supernatant was collected separately to analyze PAC. Protein pellet was suspended in PBS and its concentration was determined by BCA.

### 2.7. UPLC Analysis

UPLC Shim-Pack XR-ODS II reverse-phase column (Shimadzu; 150 × 3.0 mm i.d., 2.2 μm) was used for the analysis of PAC. Acetonitrile and water were used as a mobile phase with 0.75 mL/min flow rate. In a linear gradient elution, the concentration of acetonitrile was increased from 5 to 60% (from 1.3 to 5.1 min), to 80% (from 5.1 to 7.7. min) and 100% at 10 min and maintained till 10.9 min; the concentration was then reduced to 5% at 12 min. PAC was detected by using PDA-UV detector at 227 nm and concentration was calculated against the standard curve of PAC.

### 2.8. Mechanism of Drug Loading

Proteins in exosomes show intrinsic fluorescence due to the presence of aromatic residues of tryptophan, tyrosine and phenylalanine. This property was utilized to determine the fluorescent quenching of exosomes due to hydrophobic interaction with different concentration of PAC, as reported for human serum albumin [25]. Briefly, exosomes alone (6 mg/mL) and PAC-loaded exosomes in PBS were analyzed for fluorescent signals at excitation and emission wavelengths of 280 nm and 320 nm, respectively, using a SpectraMax Spectrofluorometer. The reduction in the fluorescent signals in the presence of PAC was calculated and suggestive that the strong hydrophobic interactions play a crucial role in drug loading onto exosomes.

### 2.9. Cell Lines and Maintenance

Human lung cancer cell lines A549 were obtained from American Type Culture Collection (Manasa, VA, USA) and taxol-resistant A549TR cells were provided by Dr. Bruce Zetter of Children’s Hospital Boston, Harvard Medical School (Boston, MA, USA). Bioware^®^ Brite Cell Line A549 Red-FLuc was procured from PerkinElmer, USA. Cells were cultured in RPMI (Gibco, Waltham, MA, USA) supplemented with 10% FBS and antibiotics (penicillin/streptomycin) at 37 °C in 5% CO_2_. No antibiotic solution was supplied to the culture media.

### 2.10. In Vitro Antiproliferative Activity

The effect of PAC and its exosomal formulation on cell viability was measured using the MTT assay. Briefly, A549 and A549TR cells were plated in 96-well plates at an initial density of 3 × 10^3^ cells per well and treated with Exo, PAC or ExoPAC and incubated for 72 h. The cell survival was determined by MTT assay, as described [26]. Briefly, A549-LUC cells (3 × 10^3^ cells/well) were plated in 96-well white plates. Cells were treated with PAC and ExoPAC at different concentrations for 72 h. Culture media were replaced with fresh media containing luciferin (150 µg/mL). The luminescence intensity was measured using a SpectraMax spectrophotometer.

### 2.11. Colony-Forming Assay

Taxol-sensitive (A549) and taxol-resistant (A549TR) cells were seeded into 6-well tissue culture plates at a density of 500 cells/well, as described [26]. The cells were treated with PAC or ExoPAC at different concentrations for 24 h. The drug-containing medium was discarded and replaced with a fresh drug-free medium. After 10 d, the plates were washed with sterile PBS, and the cells were fixed using methanol/acetic acid solution (3:1) for 5 min and stained with 0.5% crystal violet (in methanol) for 15 min. The crystal violet solution was carefully removed, the cells were rinsed with water and air dried at room temperature. The number of colonies in each well was counted manually.

### 2.12. Animal Studies

All animals were maintained according to the Institutional Animal Care and Use Committee guidelines (IACUC).

#### 2.12.1. Lung Cancer Subcutaneous Xenograft

Female athymic nude (nu/nu) mice (5–6 weeks old) were procured from Harlan (Indianapolis, IN, USA) and used to assess the antitumor efficacy. Lung tumor xenografts were produced by subcutaneously injecting human lung A549 cells (2.5 × 10^6^), in serum-free media mixed with Matrigel matrix (Becton Dickinson, Bedford, MA, USA), in the left flank of the mice. Animals were provided purified AIN93M diet and water ad libitum. Once the average tumor size reached about 100 mm^3^, mice were randomized into four groups (*n* = 10) and provided with oral doses of PBS, PAC, ExoPAC and FA-ExoPAC, three times a week. The PAC doses in all the regimens were kept equal (6 mg/kg). Tumor size, animal weights, diet intake, and overall animal health were monitored weekly. After 7 weeks of treatment, the animals were euthanized and select tissues were collected for further analysis.

#### 2.12.2. Lung Cancer Orthotopic Xenograft

##### Pilot Study

For the orthotopic lung tumor model, we first performed a pilot study to establish the effect of doses and time on tumor growth, before initiating the tumor inhibition study. After acclimation, female NOD/SCID mice (4–5-week old) were randomized into three groups (*n* = 4) and inoculated with Bioware^®^ Brite A549-Red-Fluc cells (1 × 10^6^, 2 × 10^6^, and 4 × 10^6^ cells) in 50 µL of Matrigel mixed in serum-free media (1:1; *v*/*v*) via intrathoracic injection using 30-gauge needles [27,28]; an untreated group served as control. Luciferase expressions were monitored for tumor growth twice a week. The luciferase signals were detected 15 min post-intraperitoneal injection of luciferin (120 mg/kg) by using Advanced Molecular Imager, AMI1000.

##### Tumor Inhibition Study (Low Dose)

For the tumor inhibition study, groups of female NOD/SCID mice were inoculated with A549-Red-Fluc cells (2 × 10^6^ cells) via intrathoracic injection, as described above. After 10 days, when the luminescence intensity reached approximately 6 × 10^6^ photons, animals were randomized (*n* = 10) and treated with i.v. PAC, i.v. Abraxane, p.o. ExoPAC, i.v. FA-ExoPAC or p.o. FA-ExoPAC. The i.v. doses of all regimens having PAC (6 mg/kg) were given once a week, whereas oral doses were given three times a week. The PAC and Abraxane were given i.v. to mimic the clinical scenario. Two additional groups were treated with Exo and FA-Exo. The exosome concentration in all the formulations was 50 mg/kg.

##### Tumor Inhibition—Higher Dose

This study was patterned after the low dose study and the animals were randomized (*n* = 10) and treated with PAC, Abraxane, ExoPAC and FA-ExoPAC given orally or intravenously, as described for the low dose study, except PAC was given initially at 4 mg/kg for three weeks, then switched to 8 mg/kg in all the regimens; the frequency of dosing and the exosome concentration was same as in the low-dose study. Bodyweight gains, diet intake, and overall animal physical health were monitored weekly. At euthanasia, various tissues were collected and imaged ex vivo. Lung, liver, and tumor tissues were collected and stored at −80 °C for marker analysis.

#### 2.12.3. Toxicity Study

Female C57BL/6 mice (5–6 weeks old) were purchased from Charles River Laboratories. Animals were randomized in six groups (*n* = 5) and treated with vehicle, Exo (60 mg/kg/week; oral), FA-Exo (60 mg/kg/week; oral), PAC (6 mg/kg/week; i.p.), ExoPAC (9 mg/kg/week; p.o.) and FA-ExoPAC (9 and 18 mg/kg/week; p.o.). The exosome concentration was kept constant to 60 mg/week in all the exosomal formulation-treated groups. The drug was given three times a week in all the treatment groups and continued for four weeks. Bodyweight, physical mobility and food intake were monitored twice a week throughout the study. After four weeks of treatments, animals were euthanized by CO_2_ asphyxiation. At the time of euthanasia, blood and the major organs were weighed and collected for further analysis. The spleen and femur bone were collected in fresh media to harvest the spleen and bone marrow cells.

##### Systemic Toxicity

Blood was collected at the time of euthanasia and hematological parameters were analyzed using whole blood by the CellDyn 3500 hematology analyzer (Abbott laboratories, Santa Clara, CA, USA). Serum was used to analyze the liver and kidney function enzymes, as described [23]. Electrolyte analysis was done by using an ion-selective electrode while other biochemical parameters were analyzed spectrophotometrically using AU640 Chemistry Immuno Analyzer (Beckman Coulter, Inc., Brea, CA, USA). Spleen and bone marrow were used for immune toxicity studies described below.

##### Immune Cell Analysis

Immune cell quantification was performed by staining single-cell suspensions of spleen cells with fluorescent dye-coupled antibodies to CD19 for B cells, CD5 (total T-cells), CD4, and CD8 for T-cell subsets, F4/80, CD11b, Gr-1, to identify macrophages and neutrophils, CD11c for dendritic cells, NK1.1 and CD49b for natural killer cells.

Bone marrow stem and progenitor cells were identified by negative staining for lineage-specific markers using biotin-labelled antibodies to B220, CD11b, Gr-1, CD5, CD8, Ter-119 and APC-Cy7 coupled streptavidin and positive staining for Sca-1 and c-kit. The cells stained with fluoresceinated antibodies were analyzed using an LSR II flow cytometer (BD BioSciences) and the data were analyzed using FlowJo software. All statistical analyses were performed by two-way ANOVA using group A as the control group.

For cytokine assays, spleen cells were cultured with lipopolysaccharide (LPS) or anti-CD3 antibody for 24 h in Iscove’s DMEM (IMDM) in the presence of 10% fetal calf serum. The culture supernatants were analyzed for IL-6, IL-10, IL-2 and γ-interferon using specific reagents obtained from R&D Biosystems (Minneapolis, MN, USA) and multiples reagent from MesoScale using respective recombinant cytokines as standards. Data are presented as percent control where control is the average value for spleen cells from PBS-treated mice.

### 2.13. Statistical Analysis

Statistical analysis was performed with GraphPad Prism statistical software (version 4.03; La Jolla, CA, USA) using two-way ANOVA followed by a Bonferroni post-test for xenograft studies. Data in the xenograft studies are expressed as mean ± standard error of mean (SEM) (*n* = 10). Statistical significance of differences in immune cell numbers, cytokine assays and proliferation responses between various treatments was evaluated by an unpaired Student’s *t*-test. Values of *p* < 0.05 were considered statistically significant.

## 3. Results

### 3.1. Exosome Isolation and Characterization

**Figure 1:**
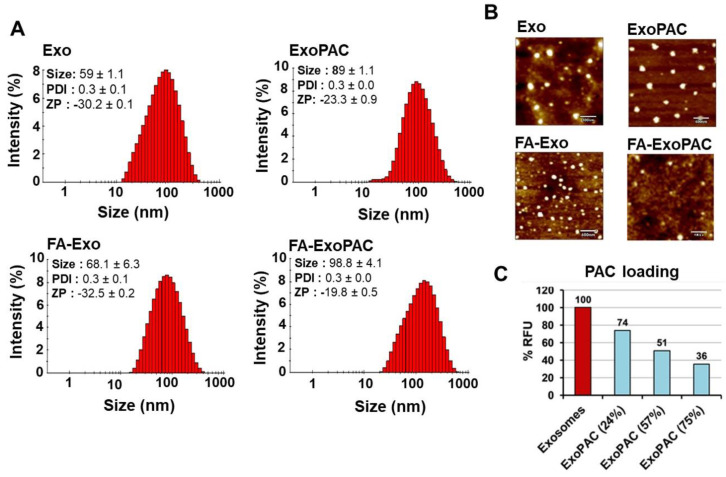
Characterization and drug loading of colostrum-derived exosomes. Size, polydispersity index (PDI), and zeta potential (ZP) of exosomes, FA-Exo, ExoPAC and FA-ExoPAC, analyzed by Zetasizer. Data represent mean ± SD from three preparations (**A**). Analysis of exosomes and ExoPAC by atomic force microscopy (AFM) after diluting with deionized water up to 10 μg/mL. For measurement, samples were placed on a silica wafer and air-dried for 30 min. AFM in tapping mode and aluminum-coated silicon probes were used for imaging (**B**). The bar diagram shows the quenching of autofluorescence from the exosomes following PAC loading (**C**). Higher quenching of fluorescence in the presence of higher drug load suggests a hydrophobic interaction of drug with exosomal proteins.

Colostrum derived exosomes are lipid bilayer nanovesicles and their diameters vary from 30–150 nm. Exosome suspension was homogenous with an average particle size of 59 ± 1.1 nm, PDI of 0.3 ± 0.1, and zeta potential of −30.2 ± 0.1 mV, as determined by Zetasizer and NanoView (Figure 1A); these analyses were performed after removing PBS from the exosomes by ultrafiltration (300,000 MWCO spin filter) since the presence of PBS increased zeta potential of the particles. Zeta view analysis of exosomes showed 0.5–1.0 × 10^14^ particles per mg of exosomal proteins. The size was confirmed with AFM (Figure 1B). Exosomes isolated from colostrum showed hallmark protein markers such as CD81, Tsg101, Alix and the anti-phagocytic protein, CD47, as described elsewhere [24].

### 3.2. Drug Loading and FA Functionalization

Exosomes were functionalized by covalently attaching FA first, followed by loading of PAC. The PAC was loaded with simple mixing of drug solution with FA-functionalized exosome. The size of exosomes was only slightly increased (68 ± 6.3 nm from 59 ± 1.1) after FA conjugation. However, PAC loading increased the size modestly for both exosomes (89 ± 1.1 from 59 ± 1.1) and FA-exosomes (98.8 ± 4.1 from 68.1 ± 6.3). The zeta potential of FA-ExoPAC (−19.8 ± 0.5) was increased compared with exosomes (−30.2 ± 0.1) and ExoPAC (−23.3 ± 0.9) (Figure 1A,B).

### 3.3. Mechanistic Understanding of Drug Loading in Exosomes

We utilized quenching of intrinsic fluorescence of surface-bound exosomal proteins to determine if the PAC was surface-bound. We observed a dose-dependent decrease in fluorescence with an increase in PAC loading to exosomes. The percent fluorescence quenching was correlated with the PAC load—a drug load of 24%, 57% and 75% resulted in 26%, 49% and 64% fluorescence quenching, respectively (Figure 1C). These data clearly suggest that at least part of the drug is sequestered in the hydrophobic domains of surface-bound exosomal proteins; however, we cannot rule out that part of the drug is in the lipid bilayer and/or lumen of the exosomes.

### 3.4. ExoPAC Inhibits Growth of Both Drug-Sensitive and Drug-Resistant Lung Cancer Cells

**Figure 2:**
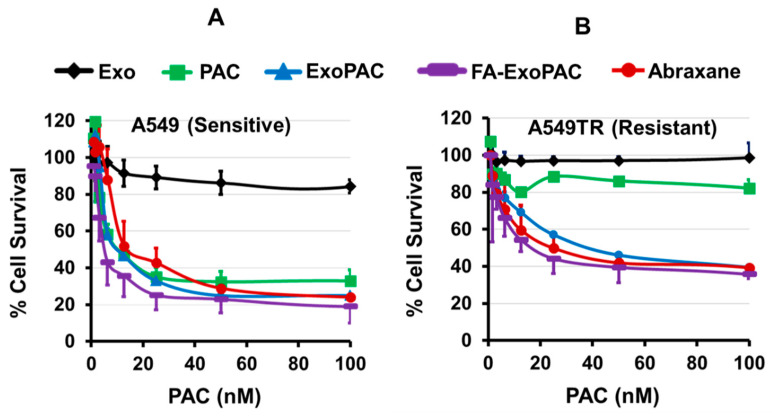
ExoPAC inhibits proliferation of drug-sensitive and drug-resistant cells. (**A**) Drug-sensitive (A549) and drug-resistant (A549TR) cells were treated with Exo, ExoPAC and FA-ExoPAC and compared with Abraxane. Antiproliferative activity was determined by MTT assay after 72 h. Exosomal PAC dose-dependently inhibited the proliferation of drug-sensitive A549 (**A**) and drug-resistant A549TR cells (**B**).

The antiproliferative effects of PAC, ExoPAC and FA-ExoPAC were determined against drug-sensitive and drug-resistant human lung cancer cells and compared with albumin-bound PAC (Abraxane). PAC and its exosomal formulations showed a dose-dependent cell growth inhibition against A549 cells. FA-ExoPAC, however, showed a twofold reduction in the IC50 values compared to PAC; the IC50 of Abraxane was similar to PAC (Figure 2A). Exosome alone (Figure 2A) and FA-Exo (data not shown) demonstrated about 20% inhibition of A549 cells. To determine if the exosomal formulation could chemosensitize the drug-resistant cells, we tested all the formulations against taxol-resistant A549TR lung cancer cells. PAC did not show any inhibition of the resistant cells up to 200 nM. However, the data indicated that FA-ExoPAC was able to inhibit the growth of the drug-resistant cells dose-dependently with the IC50 values of 12.5 nM. ExoPAC and Abraxane showed a similar effect. (Figure 2B).

### 3.5. Colony Formation Assay

**Figure 3:**
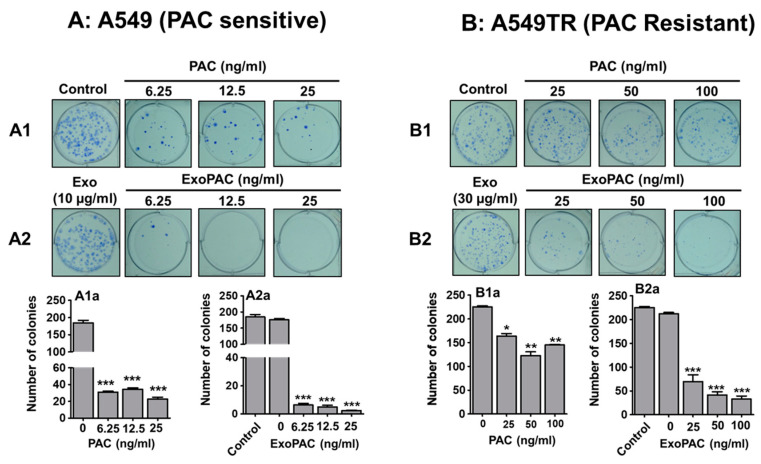
Exosomal PAC inhibited colony formation in NSCLC cells. Representative images showing the colony formation assay in drug-sensitive A549 (**A**) and drug-resistant A549TR (**B**) cells. Lung cancer cells were seeded (500 cells/well) in a six-well plate and incubated with different concentrations of PAC and ExoPAC. After 10 days, developed colonies were fixed, stained and counted manually. While PAC was effective, only against drug-sensitive cells (**A1**,**B1**), ExoPAC shows dose-dependent inhibition of colony formation of both sensitive and resistant cells (**A2**,**B2**). Statistical analysis was performed using the Student’s *t*-test. *, *p* < 0.05; **, *p* < 0.01; ***, *p* < 0.001.

To validate the observed antiproliferative effects, we investigated the potential effects of PAC and ExoPAC on the replicative ability of drug-sensitive (A549) and its drug-resistant variant (A549TR) using colony formation assay (Figure 3A,B). Similar to the MTT data, while PAC showed 65% inhibition of colony formation at 6.25 nM, ExoPAC had over 90% inhibition at the same dose. Interestingly, the effect of PAC (25–100 nM) was minimal on the resistant cells, while ExoPAC had significant inhibition starting from 25 nM. As expected, ExoPAC showed dose-dependent inhibition of colony formation against both A549 (Figure 3A; Supplementary Figure S1) and A549TR (Figure 3B; Supplementary Figure S2) cells; ExoPAC inhibited colony formation in both cell lines greater than PAC alone.

### 3.6. Antitumor Efficacy Following Oral Administration of ExoPAC

#### 3.6.1. Subcutaneous Lung Tumor Xenografts

**Figure 4:**
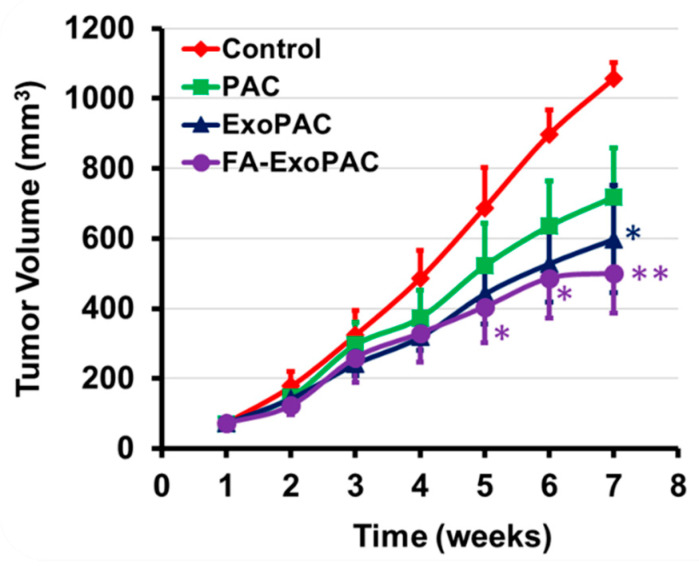
Antitumor activity against subcutaneous xenografts. Following inoculation with A549 cells, nude mice were treated with oral gavage three times a week with PAC (6 mg/kg bw), ExoPAC and FA-ExoPAC (6 mg PAC and 50 mg Exo protein/kg bw). Data represent average ± SD of means (*n* = 8). Statistical analysis was performed using the Student’s *t*-test. * *p* < 0.05; ** *p* < 0.01.

We determined the antitumor efficacy of ExoPAC and FA-ExoPAC using athymic nude mice bearing subcutaneous A549 xenografts and compared them with PAC. There was no difference in the body weight or diet consumption, suggesting no gross toxicity due to PAC or ExoPAC. Compared to untreated control, PAC (6 mg/kg) showed about 30% but statistically insignificant inhibition of the tumor growth. However, ExoPAC (6 mg/kg PAC and 50 mg exosomal proteins/kg) showed a significant (45%; *p* < 0.05) growth inhibition at the end of the study (Figure 4). FA-ExoPAC at the same dose was even more effective (54%; *p* < 0.05) with the growth inhibition occurring as early as five weeks after the treatment (Figure 4).

#### 3.6.2. Orthotopic Lung Tumor Xenografts

**Figure 5:**
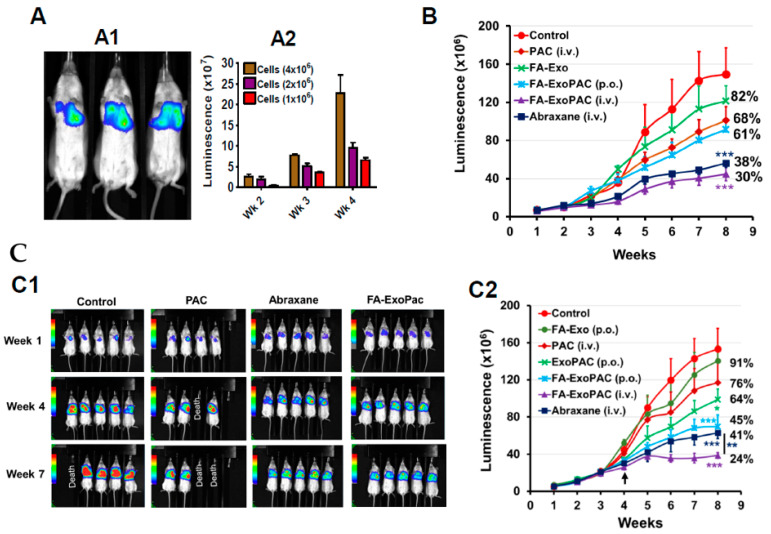
Antitumor activity against orthotopic xenografts. Detection of orthotopic lung cancer using bioluminescent A549-Red-luc cells. (**A**) A1: image of live animals after 27 d of inoculation with 2 × 10^6^ cells (3 mice/group). A2: Mean of bioluminescence signals. (**B**) Inhibition of A549 orthotopic lung tumors in NOD Scid female mice (*n* = 10) by i.v. paclitaxel (PAC), i.v. Abraxane, and *p.o.* ExoPAC and *p.o.* FA-ExoPAC (three doses weekly), all given at 6 mg/kg. FA-Exo was used as control for FA-ExoPAC. (**C**) Inhibition of A549 orthotopic lung tumors in NOD Scid female mice (*n* = 10) by i.v. PAC, Abraxane, FA-ExoPAC (once weekly) and *p.o.* ExoPAC and FA-ExoPAC (three doses weekly), all given at 4 mg/kg until three wks, then switched to 8 mg/kg, as indicated by an arrow in C2. Representative images of animals at different time points in the indicated treatment groups (C1) and time-dependent tumor inhibition (C2). Statistical analysis was performed using the Student’s *t*-test. *, *p* < 0.05; **, *p* < 0.01; ***, *p* < 0.001.

We first established an orthotopic lung tumor model in a pilot study using Bioware^®^ Brite A549 Red-FLuc lung cancer cells. Live animal imaging showed the bioluminescence signals from lung tumors (Figure 5A); tumor growth was dose- and time-dependent (Figure 5A). The tumors could be detected as early as 10–12 days after tumor cell inoculation, with nearly exponential growth. After four weeks, animals inoculated with different cell numbers showed a dose-dependent increase in bioluminescence signals. Based on the tumor growth and expected tumor size, we used 2 × 10^6^ cells in efficacy studies.

In an efficacy study, two doses of PAC (6 mg/kg and 8 mg/kg) were tested. Figure 5B shows the data from the low-dose study; i.v. PAC showed 32% inhibition of tumor growth whereas *p.o.* FA-ExoPAC showed a somewhat higher growth inhibition (39%), although the difference was not statistically significant; the nonfunctionalized formulation (ExoPAC) showed only a slight inhibition (18%). However, FA-ExoPAC administered i.v. resulted in significantly higher growth inhibition (70%; *p* < 0.001) matching the efficacy of i.v. Abraxane (62%; *p* < 0.001).

The data presented in Figure 5(C1,C2) demonstrates significant tumor growth inhibition in the following order: i.v. FA-ExoPAC (76%; *p* < 0.001) > p.o. FA-ExoPAC (55%, *p* < 0.001) ≈ i.v. Abraxane (59%, *p* < 0.001) > *p*.o. ExoPAC (36%, *p* < 0.05) > i.v. PAC (24%) > p.o. FA-Exo (9%).

Clearly, oral FA-ExoPAC far exceeded the efficacy of i.v. PAC and, in fact, matched the efficacy elicited by i.v. Abraxane; i.v. FA-ExoPAC exceeded the efficacy of i.v. Abraxane. Dose-optimization studies are warranted to identify the most efficacious oral doses and frequency of FA-ExoPAC formulation. At the completion of the study, we observed that animals treated with i.v. FA-ExoPAC (6 and 8 mg/kg) did not exhibit any mortality, while about 42% of the animals died in solvent-based i.v. PAC and 30% in control groups. Importantly, ExoPAC-treated animals also showed a significantly improved overall health index compared to PAC or untreated controls (Supplementary Figure S3).

### 3.7. Assessment of Toxicity Due to PAC and ExoPAC

#### 3.7.1. Systemic Toxicity

For the analysis of the potential toxicity of PAC, exosomes and ExoPAC, wild-type C57BL/6 mice were treated for 28 days and assessed for gross and systemic toxicity. We observed no difference in the body weight, diet intake and physical wellness of treated versus control animals. We analyzed the levels of liver enzymes (aspartate transaminase, alanine aminotransferase, alkaline phosphatase, gamma-glutamyl transpeptidase, amylase, and lipase) in the serum of animals treated either with PAC, exosomes or ExoPAC. PAC significantly changed the levels of amylase and total bilirubin. These effects were not evident with ExoPAC suggestive of hepatoprotective role when PAC was embedded in exosomes. Similarly, toxicity caused by PAC in kidney function tests and hemopoietic parameters was mitigated by its formulations in exosome (Table 1 and Table 2).

#### 3.7.2. Immunotoxicity

**Figure 6:**
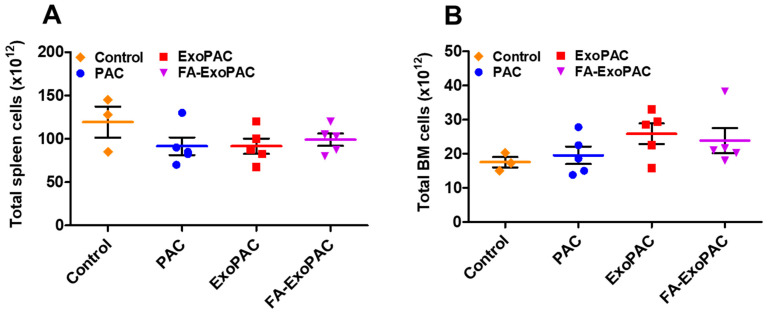
Potential immunotoxicity of PAC and FA-ExoPAC. Female C57BL/6 mice were treated with Exo, PAC and FA-ExoPAC for four weeks. At euthanasia, spleen and bone marrow cells were collected. Live splenic (**A**) and bone marrow (**B**) cell counts were performed by trypan blue exclusion.

**Figure 7:**
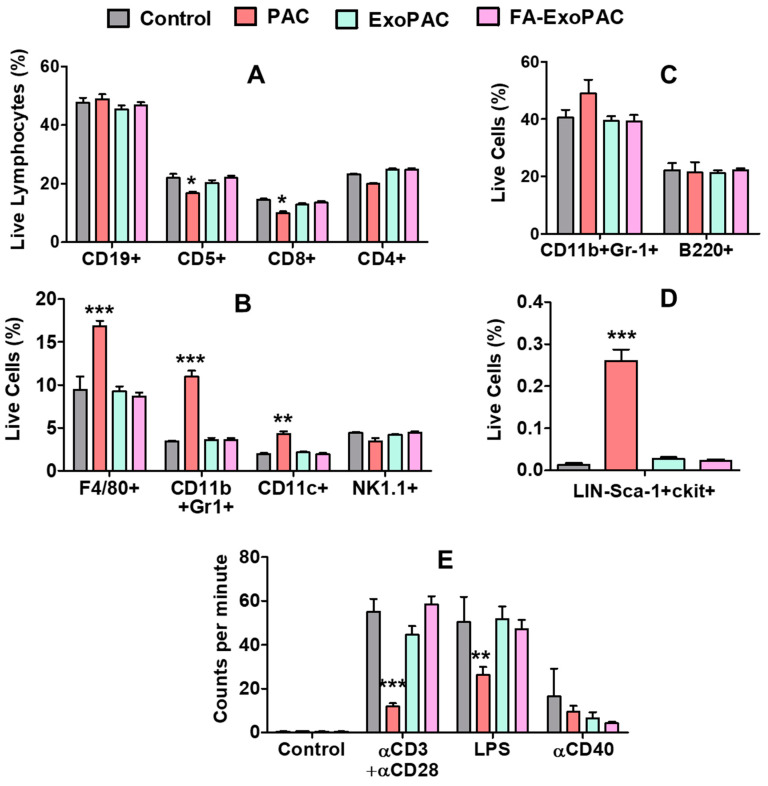
Potential immunotoxicity of PAC and FA-ExoPAC. Female C57BL/6 mice were treated with Exo, PAC and FA-ExoPAC for four weeks. At euthanasia, spleen and bone marrow cells were collected. Splenic cells were stained with multiple fluorochrome-conjugated antibodies. Samples were run using LSRII cytometer and data was analyzed by FlowJo software. Effect of treatment was analyzed on B cells, total T-cells, T helper cells, and cytotoxic T-cells (**A**), macrophages, neutrophils and dendritic cells (**B**), B cell (**C**), %LSK cells (**D**) and T-cell proliferation (**E**). All statistical analysis was performed by two-way ANOVA and compared with untreated control. *, *p* < 0.05; **, *p* < 0.01; ***, *p* < 0.001.

Single cell suspensions of splenic and bone marrow cells were prepared and viable cells were quantified by trypan blue exclusion. No significant differences were found in the total cell numbers of splenic as well as bone marrow cells (Figure 6A,B) upon any of the treatments. Next, splenic cells were stained with multiple fluorochrome-conjugated antibodies specific to B cells (CD19), total T-cells (CD5), T-cell subsets (CD4, CD8) and were analyzed by flow cytometry. Overall percentages of the different lymphocyte subsets were not significantly different between control and ExoPAC or FA-ExoPAC treatment groups. Interestingly there were small reductions in total CD5+ and CD8+ T-cells in the spleen after treatment with PAC alone but these reductions were abrogated when PAC was provided as ExoPAC or FA-ExoPAC. On the other hand (Figure 7B). PAC caused a significant increase (*p* < 0.001) of macrophages (F4/80), neutrophils (CD11b+Gr-1+) and dendritic cells (CD11c+) compared to control, which were mitigated by the use of exosomal formulations. Natural killer cells (NK1.1+) were unaffected irrespective of treatment (Figure 7C). However, in the analysis of bone marrow cells, there was no difference in neutrophils (CD11b+Gr-1+) and B cells (B220) by any treatment (Figure 7C).

Stem and progenitor (LSK or LIN-Sca-1+cKit+) cells were highly increased by treatment with PAC (*p* < 0.001), whereas this increase was not found with ExoPAC formulations suggesting that exosomal formulation could mitigate the toxicity associated with PAC (Figure 7D). Splenic cells were induced to proliferate by treating with different stimulants for 72 h, which is an important requirement for effective immune response to tumor cells or pathogens. Cells were pulsed with 3H-thymidine for 4 h, then, cells were harvested and the incorporation of radioactivity was quantified using a beta-plate counter. PAC treatment showed a lower T-cell proliferation and a lower T-cell independent B-cell proliferation induced by LPS (*p* < 0.01). However, exosomal formulation mitigated these adverse effects. There was no significant difference in the αCD40 treatment, which represents a T-cell-dependent B-cell proliferation response (Figure 7E).

**Figure 8:**
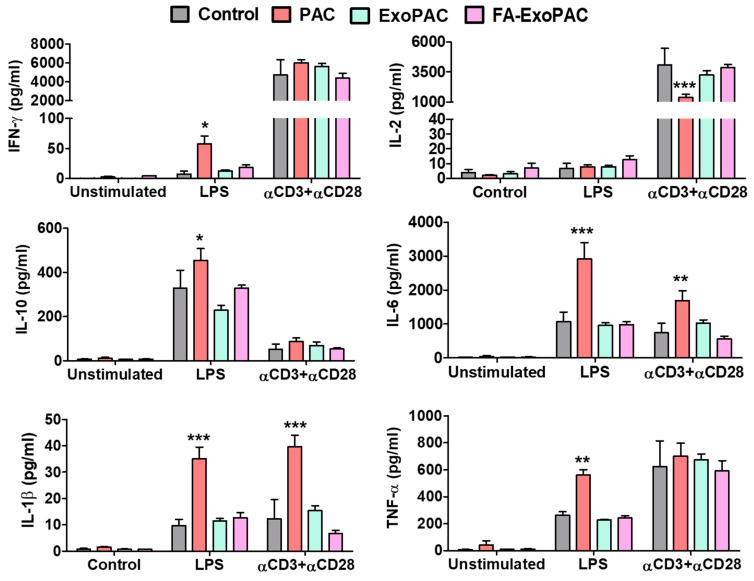
Potential immunotoxicity of PAC and FA-ExoPAC. Female C57BL/6 mice were treated with Exo, PAC and FA-ExoPAC for four weeks. At euthanasia, spleen and bone marrow cells were collected. Splenic cells were treated with the different stimulant for 24 h. Sups were collected and MesoScale analysis (V-PLEX of 6 cytokines) was performed. LPS was used for B-cell response and αCD3+αCD28 for T-cell response. All statistical analysis was performed by two-way ANOVA and compared with untreated control. *, *p* < 0.05; **, *p* < 0.01; ***, *p* < 0.001.

In order to assess cytokine response, splenic cells were treated with different stimulants for 24 h. Sups were collected and MesoScale analysis (V-PLEX of 6 cytokines) was performed. LPS was used for B-cell and macrophage response and αCD3+αCD28 was used for T-cell response (Figure 8). We observed increase in almost all cytokines (except IL-2, which was decreased) in response to PAC treatment, which was mitigated with the exosome formulations suggesting protection of PAC-induced immunotoxicity by exosomal formulations (Figure 8).

## 4. Discussion

PAC is an antineoplastic chemotherapeutic drug that is routinely used as the first- or second-line chemotherapeutic in the treatment of a broad spectrum of human cancers, including lung cancer. As with many other chemo drugs, PAC exhibits poor oral bioavailability; hence, it is administered intravenously. To increase bioavailability, several drug delivery formulations of PAC have been developed, including nanoparticle albumin-bound (Abraxane^®^), liposomal (Lipusu^®^), polymeric micelles (Genexol^®^ PM), polymeric-drug conjugates (Xyotax™/OPAXIO) and an injection concentrate for nanodispersion (Taclantis™/Bevetex^®^), as reviewed by Chor et al. [8]. Clinical translatability of these nanoformulations was impeded due to various factors like toxicity, scalability and cost. In addition to cremophor-based PAC, Abraxane is the only formulation of PAC approved by the FDA to date while the remaining formulations, also as i.v. therapeutics, are currently in clinical trials at various stages. In a randomized multinational phase 3 study (NCT02594371) lead by Athenex Inc., an oral formulation of PAC and Encequidar was evaluated in women with metastatic breast cancer. This combination therapeutic showed improved progression-free survival and overall survival compared to i.v. PAC in breast cancer patients [29]. Encequidar, although not systemically absorbed, is an inhibitor of multidrug resistance efflux pump P-glycoprotein that increases the oral bioavailability of PAC by preventing the efflux of PAC from intestinal epithelial cells in the GI tract. While oral PAC/encequidar carried less risk of neuropathy and alopecia compared to i.v. PAC, higher risk for GI and neutropenia adverse events was found [30]. These results exemplify the potential of oral PAC for the treatment of cancer while mitigating, in-part, toxicity of bolus i.v. dosing, however, the oral PAC formulation used in these clinical studies lacks specificity.

The primary objective of this study is to develop a tumor-targeted oral formulation of PAC (FA-ExoPAC) to improve the overall efficacy and safety profile while providing a user-friendly, cost-effective alternative to bolus i.v. PAC. Exosomes provide a nontoxic scalable and cost-effective approach to drug delivery. Exosomes are biogenic nanocarriers and have been shown by us and others to deliver both small and macromolecules to the tumor site [19,21,22,23,24]. Most of the current exosomal delivery technologies rely on harvesting exosomes from cells grown in high-density bioreactors. Mendt et al. [31] reported production of 10–15 × 10^12^ exosomes per bioreactor culture. In comparison, exosomes isolated from milk or colostrum are abundant in a readily available source, bovine milk, which dominates commercial production and is estimated to be 85% of worldwide milk consumption [32]. Bovine milk contains abundant exosomes (239 ± 9.6 mg exosomal protein or 33 × 10^16^ particles/L) [22]. The abundance of exosomes is further increased in bovine colostrum. Clearly, bovine milk/colostrum contains several orders of magnitude higher amounts of exosomes/L than media from high-density bioreactors.

The generally recognized safety of colostrum powder combined with high exosome yield makes it a biocompatible source for cost-effective, large-scale production of exosomes. In these studies, bovine colostrum powder derived exosomes showed small uniform distribution of size, approximating 60 nm, which only slightly increased (13%) following the covalent attachment of the tumor-targeting ligand FA while a modest increase (approx. 30%) in exosomal size was observed following the loading of PAC. This modest increase in exosomal size and our observed fluorescent quenching of exosome surface-bound proteins due to hydrophobic interaction with increasing concentrations of PAC could be attributed to sequestration of PAC by the hydrophobic domains of the exosomal surface-bound proteins; however, we cannot rule out that part of the drug is in the lipid bilayer and/or lumen of the exosomes.

Cancer cells develop resistance to PAC through several mechanisms like initiating the efflux pump, DNA mutations and changes in microtubule dynamics. In our in vitro antiproliferative and colony-forming studies, we noted while PAC is effective against the drug-sensitive cells, its exosomal formulations showed significant activity even against drug-resistant cells, suggesting a role for exosomes in preventing the efflux of PAC. The present study suggests that lung cancer drug resistance towards PAC could be avoided by using exosomal formulation. We have previously shown that the growth of normal epidermal keratinocytes (HEKn) and Beas-2B epithelial cells was unaffected by milk exosomes [21,22]. In the present study we showed that ExoPAC did not disturb the immune homeostasis which was affected by PAC alone in altering some immune cell subsets. Also, ExoPAC, unlike PAC did not alter the cytokine production or growth response of various immune cells further attesting to near absence of any immunotoxicity.

Tumor-targeted drug delivery approaches have attracted extensive attention due to their ability to achieve higher drug accumulation in tumor site and reduce off-target effects. Under physiological conditions, reduced form of folates transports into the cells via reduced folate carrier (RFC) through an anion-exchange mechanism. After entering the cell, folate plays a crucial role in biosynthesis of building blocks of DNA synthesis, methylation and repair [33]. The other form of folate entry into the cell is through folate receptor (FR). There are four isoforms of FR (FRα, FRβ, FRγ and FRδ) identified in humans. In cancer cells, the expression of FRα covers the entire cell surface due to loss of its polarized cellular location. FRα, the target of FA, is present at low levels in normal tissues but it is overexpressed in majority of NSCLCs and in lung adenocarcinomas [34]. Our data show >100-fold higher expression levels in lung tumors versus normal lung. We also showed that FA-Exo-AF750 resulted in significantly higher tumor accumulation of exosomes compared with nonfunctionalized Exo-AF750 [24]. Thus, the limited expression and restricted distribution pattern of this receptor make it attractive for targeting lung tumors [35]. Once FA or RFC binds to the FR receptor, the total complex enters into the cells via the process of endocytosis. In this context, FA serves as a feasible option to direct ExoPAC to cancer cells. In this study, FA was covalently attached to exosomes to enhance the specificity of ExoPAC. Since FA is not retained in the kidneys, no significant toxicities have been observed in rodent models or humans with FRα-targeted agents [35,36,37].

Oral dosing of chemotherapeutics offers many advantages—including flexibility of timing, location of administration, flexibility of drug exposure, reduction of the use of the healthcare resources for in-patient and ambulatory-patient care services, and a better quality of life [11,12]. However, due to the poor GI absorption and hepatic first-pass effect, bolus doses of PAC and other chemotherapeutics are required for efficacy and likely contribute to overall toxicity.

It is evident from our previous study that ExoPAC using exosomes from bovine milk exhibits enhanced anticancer response versus free PAC against lung cancer cells and efficiently inhibits the lung cancer subcutaneous xenografts [19]. In this study, first we show that given orally, ExoPAC and FA-ExoPAC, using exosomes isolated from bovine colostrum powder, demonstrate much higher activity compared to free PAC in subcutaneous lung cancer xenografts, followed by efficacy studies using an orthotopic model to mimic relevant tumor microenvironment. Our previous studies demonstrate that exosomes maintain their integrity in gastrointestinal pH and the release of PAC was consistent at wide ranges of pH (5, 5.8 and 6.8) resembling physiological conditions of the body, suggesting that ExoPAC formulations are stable in the harsh environment of GI and produce higher activity compared to free PAC.

As per our previous studies, colostrum exosomes express CD47 protein marker along with other hallmark exosomal proteins on their surface, which enhances the circulatory half-life of the exosomal drug formulations [24]. Further, FA functionalization on exosomes leads to trafficking to tumor site due to the presence of folate receptors (FR-α) and reduced folate receptors (RFC) on tumor cells. At the tumor site, exosomes are internalized in the cancer cells through several mechanisms like endocytosis, phagocytosis, micropinocytosis and/or fusion with cellular plasma membrane [38,39,40,41]. After entering the cell cytoplasm, exosomes directly release their payloads or undergo lysosomal digestion to release the drug contents [42]. In this study, we postulated that FA-ExoPAC releases PAC inside the cancer cells either through direct release or by the lysozyme-mediated digestion, which was clearly demonstrated by its in vivo antitumor response against subcutaneous and orthotropic lung cancer xenografts.

The orthotopic xenograft models represent a clinically relevant tumor model with respect to the tumor’s primary site, microenvironment and metastasis [43,44]. These models are further improved with the advent of imaging techniques, which help in the measurement of internally implanted orthotopic tumors. In this study, using an orthotopic model, we demonstrate that FA-ExoPAC given either i.v. or p.o. has much greater efficacy compared to PAC i.v. and that for the higher dose study, FA-ExoPAC given orally produced efficacy similar to i.v. Abraxane. Noteworthy is that when used i.v., FA-ExoPAC produced significantly higher antitumor activity compared to Abraxane, suggesting the potential of exosome-based drug formulation for the management of cancer. The enhanced activity of FA-ExoPAC could be due to tumor targeting, slow release of PAC from the exosomes and intrinsic ability of exosomes to inhibit the cancer cells [21].

Clinical translatability of new drugs or nanoformulations are often limited due to toxicity concerns at various stages of drug discovery and development. While efficacious, PAC is generally not well tolerated and its limitations include low solubility, and significant toxicity associated with both the drug and the solvent (Cremophor EL), including hypersensitivity reactions, bronchospasms, hypotension, hematological toxicity, peripheral sensory neuropathy, myalgia, arthralgia and alopecia [8,45]. We have demonstrated FA-ExoPAC clearly enhanced therapeutic efficacy of PAC diminishing the dose-related toxicity issues. Our previous toxicity study reports establish milk exosomes as nontoxic and nonimmunogenic [19]. The present study using colostrum exosomes further supports lack of PAC-related gross, systemic and immune toxicity concerns when used in an exosomal formulation.

## 5. Conclusions

In summary, our findings have potential clinical implications for the management of advanced non-small cell lung cancer and potentially other cancers routinely treated with PAC. We showed that: (i) an abundance of exosomes in standardized bovine colostrum powder displays high PAC loading, and enhanced tumor targeting with FA-functionalized exosomes; (ii) PAC in exosomal formulation exhibits strong activity against both drug-sensitive (A549) and drug-resistant (A549TR) lung cancer; (iii) ExoPAC administered orally inhibit both subcutaneous and orthotopic lung tumors, and the efficacy is enhanced when FA-functionalized exosomes are used; (iv) p.o. FA-ExoPAC surpassed the efficacy of i.v. PAC and matched efficacy of i.v. Abraxane in one study; (v) i.v. FA-ExoPAC exceeded efficacy of i.v. Abraxane, the only FDA-approved albumin-bound nanoformulation of PAC; and (vi) FA-ExoPAC minimally perturbed the immune homeostasis of the host, thus eliminating potential adverse effects of PAC on the immune system. Together, these data provide a strong rationale for the development of oral exosomal formulations of PAC as a therapeutic alternative to current therapies.

## Figures and Tables

**Table 1:** Effect on biochemical profile (systemic toxicity) following 28 days exposure to Exo, PAC and ExoPAC in C57BL/6 mice.

Parameter	Control	Exo	PAC	ExoPAC	FA-ExoPAC
Liver Profile					
AST (SGOT)	400 ± 220	343 ± 102	381 ± 80	314 ± 73	423 ± 83
ALT (SGPT)	39.7 ± 20.0	56.0 ± 19.5	51.8 ± 20.2	42.0 ± 4.9	53.2 ± 17.6
Alk Phosphatase	62.8 ± 62.2	89.2 ± 17.4	10.5 ± 4.9	14.0 ± 9.9	63.0 ± 49.5
GGT	1.0 ± 0.0	1.0 ± 0.0	1.0 ± 0.0	1.0 ± 0.0	1.0 ± 0.0
Amylase	378 ± 164	523 ± 17	987 ± 606 *	525 ± 28	476 ± 80
CPK	1044 ± 499	1488 ± 596	1231 ± 541	1030 ± 393	1313 ± 278
Total Bilirubin	0.6 ± 0.4	0.1 ± 0.0 *	0.1 ± 0.0 *	0.5 ± 0.3 ^#^	0.6 ± 0.3 ^##^
Kidney Function Test					
BUN	16.6 ± 4.1	19.6 ± 0.9	12.6 ± 3.1	16.8 ± 3.8	15.4 ± 3.1
Creatinine	0.2 ± 0.0	0.2 ± 0.0	0.2 ± 0.0	0.2 ± 0.0	0.2 ± 0.0
BUN/CreatRatio	83.1 ± 20.5	98.0 ± 4.5	63.0 ± 15.7	84.0 ± 19.2	77.0 ± 15.7
Phosphorus	13.0 ± 4.8	18.5 ± 4.6	10.4 ± 0.6	10.1 ± 0.8	10.1 ± 0.8
Calcium	7.5 ± 1.7	11.0 ± 0.8 ***	8.7 ± 0.6	8.4 ± 0.5	8.1 ± 0.4
Magnesium	5.0 ± 2.0	4.2 ± 0.3	3.8 ± 0.8	3.9 ± 0.4	4.2 ± 0.5
Sodium	120.5 ± 23.5	150.2 ± 5.7 *	137.2 ± 6.3	135.8 ± 6.3	131.6 ± 5.9
Potassium	17.5 ± 15.5	9.2 ± 0.8	9.0 ± 2.2	10.4 ± 1.3	12.5 ± 1.9 ^#^
NA/K Ratio	10.6 ± 5.6	16.4 ± 1.8 *	16.0 ± 2.6	13.6 ± 1.9	10.8 ± 2.5 ^#^
Chloride	115.4 ± 13.9	111.8 ± 8.0	121.8 ± 3.8	123.2 ± 3.8	117.6 ± 5.9
Total Protein	7.9 ± 3.7	6.2 ± 0.5	5.6 ± 1.3	6.4 ± 0.6	6.7 ± 0.6
Albumin	4.9 ± 2.1	3.7 ± 0.3	2.9 ± 0.8	3.8 ± 0.6	4.2 ± 0.5 ^#^
Globulin	2.8 ± 1.0	2.4 ± 0.3	2.7 ± 0.6	2.7 ± 0.3	2.5 ± 0.4
A/G Ratio	1.8 ± 0.4	1.6 ± 0.2	1.1 ± 0.1 **	1.5 ± 0.4	1.7 ± 0.3 ^##^
Cholesterol	343 ± 317	142 ± 20	148 ± 44	157 ± 31	204 ± 47
Triglyceride	60.6 ± 29.8	84.6 ± 14.3	93.8 ± 16.9 *	84.0 ± 9.9	64.4 ± 10.4 ^#^
Glucose	147.1 ± 40.4	89.8 ± 66.8	109.2 ± 17.6	154.0 ± 7.0 ^#^	159.6 ± 26.8 ^##^

Female C57BL/6 mice (5–6 weeks old) were provided control diet (AIN 93M) and water ad libitum and treated with colostrum-derived exosomes (60 mg/kg, b. wt.) by oral gavage, i.p. PAC (9 mg/kg) and ExoPAC and FA-ExoPAC (9 mg/kg PAC and 60 mg/kg exosome) for 28 days, three times a week. At euthanasia, blood was collected and analyzed using an automated AU640 Chemistry Analyzer by Antech diagnostics. Data represent average ± SD of four animals. Statistical analysis was performed using the Student *t*-test. Asterisks represent comparison to control while # represents a comparison to PAC group. *, *p*-value < 0.05; **, *p* < 0.01; ***, *p* < 0.001; #, *p*-value < 0.05; ##, <0.01.

**Table 2:** Effect on hematological parameters (systemic toxicity) following 28-day exposure to exosomes, PAC and ExoPAC in C57BL/6 mice.

Parameter	Control	Exo	PAC	ExoPAC	FA-ExoPAC
WBC	7.4 ± 1.6	4.3 ± 1.9 **	7.7 ± 0.9	5.3 ± 1.7 *^,#^	4.6 ± 2.0 *^,#^
RBC	9.2 ± 0.4	9.0 ± 0.4	7.8 ± 0.5 ***	8.0 ± 2.3	8.5 ± 0.9
HGB	14.5 ± 0.6	14.7 ± 0.5	12.8 ± 0.4 ***	12.4 ± 4.3	13.3 ± 1.7
HCT	45.7 ± 1.4	46.5 ± 2.1	37.2 ± 2.6 ***	38.6 ± 11.9	40.4 ± 4.2 **
MCV	49.3 ± 1.7	51.3 ± 0.5	47.4 ± 1.1	48.0 ± 1.9	47.2 ± 0.8 *
MCH	15.8 ± 0.9	16.3 ± 0.2	16.5 ± 0.7	15.1 ± 1.7	15.6 ± 0.9
MCHC	32.0 ± 2.1	31.8 ± 0.5	34.6 ± 1.9	31.6 ± 2.8	33.0 ± 1.9
Platelet Count	856 ± 111	829 ± 145	891 ± 131	674 ± 260	570 ± 202 **^,#^
Neutrophils	9.7 ± 3.8	13.5 ± 6.1	30 ± 11.6 ***	8.8 ± 2.8 ^##^	10.8 ± 2.4 ^##^
Bands	0.0 ± 0.0	0.0 ± 0.0	0.0 ± 0.0	0.0 ± 0.0	0.0 ± 0.0
Lymphocytes	87.1 ± 3.1	82.3 ± 4.2	66 ± 12.6 ***	87.6 ± 3.0 ^##^	84.8 ± 3.8 ^##^
Monocytes	1.1 ± 1.3	3.0 ± 2.3	1.0 ± 0.0	1.0 ± 0.0	1.0 ± 0.0
Eosinophils	2.0 ± 1.2	1.3 ± 0.5	3.0 ± 1.2	2.6 ± 0.9	3.4 ± 1.7
Basophils	0.0 ± 0.0	0.0 ± 0.0	0.0 ± 0.0	0.0 ± 0.0	0.0 ± 0.0
Absolute Neutrophils	688.1 ± 208.5	561.3 ± 314.7	2345 ± 1026 **	453.2 ± 170.1 ^##^	479.6 ± 221.1 ^##^
Absolute Lymphocytes	6519 ± 1563	3546 ± 1686	5008 ± 1082	4664 ± 1573	3942 ± 1731 *
Absolute Monocytes	44.0 ± 17.0	115.8 ± 79.4	76.6 ± 8.7	53.0 ± 16.8 ^#^	46.0 ± 19.5 ^#^
Absolute Eosinophils	152.0 ± 101.4	52.3 ± 24.8 *	231 ± 105	130.0 ± 37	132.0 ± 22

Female wild-type C57BL/6 mice (5–6 weeks old) were provided control diet (AIN 93M) and water ad libitum and treated with colostrum-derived exosomes (60 mg/kg, b. wt.) by oral gavage, i.p. PAC (9 mg/kg) and ExoPAC and FA-ExoPAC (9 mg/kg PAC and 60 mg/kg exosome) for 28 days, three times a week. At euthanasia, blood was collected and analyzed using an automated AU640R Chemistry Analyzer by Antech diagnostics. Data represent average ± SD of four animals. Statistical analysis was performed using the Student *t*-test. *, *p* < 0.05; **, *p* < 0.01; ***, *p* < 0.001 in comparison to control group. #, *p* < 0.05 and ##, *p* < 0.01 in comparison to PAC group.

## Data Availability

Not applicable.

## Supplementary Materials

The following are available online at https://www.mdpi.com/2072-6694/13/15/3700/s1, Figure S1: Inhibition of A549 colony formation by PAC and ExoPAC, Figure S2: Inhibition of resistant A549TR colony formation by PAC and ExoPAC, Figure S3: Health index of the animals treated with PAC and ExoPAC.
