# Correction: Kandimalla et al. Targeted Oral Delivery of Paclitaxel Using Colostrum-Derived Exosomes. *Cancers* 2021, *13*, 3700

**DOI:** 10.3390/cancers17182955

**Published:** 2025-09-10

**Authors:** Raghuram Kandimalla, Farrukh Aqil, Sara S. Alhakeem, Jeyaprakash Jeyabalan, Neha Tyagi, Ashish Agrawal, Jun Yan, Wendy Spencer, Subbarao Bondada, Ramesh C. Gupta

**Affiliations:** 1 James Graham Brown Cancer Center, University of Louisville, Louisville, KY 40202, USA; raghuram.kandimalla@louisville.edu (R.K.); farrukh.aqil@louisville.edu (F.A.); neha.tyagi@louisville.edu (N.T.); ashish.phe@iitbhu.ac.in (A.A.); jun.yan@louisville.edu (J.Y.); 2 Department of Pharmacology and Toxicology, University of Louisville, Louisville, KY 40202, USA; 3 Department of Medicine, University of Louisville, Louisville, KY 40202, USA; 4 Department of Microbiology, Immunology & Molecular Genetics, University of Kentucky, Lexington, KY 40536, USA; sara.alhakeem@bioagilytix.com (S.S.A.); subbarao.bondada@uky.edu (S.B.); 5 3P Biotechnologies, Inc., Louisville, KY 40202, USA; jp3pbiotech@gmail.com (J.J.); wendyspencer3p@gmail.com (W.S.); 6 Department of Surgery, University of Louisville, Louisville, KY 40202, USA

## Figure and Table Legend

In the original publication [1], there was a mistake in the legends for Tables 1 and 2. The original table legends in the article indicated the doses of PAC, ExoPAC, and FA-ExoPAC as 8 mg/kg; however, the correct dosage was 9 mg/kg. The correct legends appear below.

Correct Table 1 legend:

Female C57BL/6 mice (5–6 weeks old) were provided control diet (AIN 93M) and water ad libitum and treated with colostrum-derived exosomes (60 mg/kg, b. wt.) by oral gavage, i.p. PAC (9 mg/kg) and ExoPAC and FA-ExoPAC (9 mg/kg PAC and 60 mg/kg exosome) for 28 days, three times a week. At euthanasia, blood was collected and analyzed using an automated AU640 Chemistry Analyzer by Antech diagnostics. Data represent average ± SD of four animals. Statistical analysis was performed using the Student *t*-test. Asterisks represent comparison to control while # represents a comparison to PAC group. *, *p*-value < 0.05; **, *p* < 0.01; ***, *p* < 0.001; #, *p*-value < 0.05; ##, <0.01.

Correct Table 2 legend:

Female wild-type C57BL/6 mice (5–6 weeks old) were provided control diet (AIN 93M) and water ad libitum and treated with colostrum-derived exosomes (60 mg/kg, b. wt.) by oral gavage, i.p. PAC (9 mg/kg) and ExoPAC and FA-ExoPAC (9 mg/kg PAC and 60 mg/kg exosome) for 28 days, three times a week. At euthanasia, blood was collected and analyzed using an automated AU640R Chemistry Analyzer by Antech diagnostics. Data represent average ± SD of four animals. Statistical analysis was performed using the Student *t*-test. *, *p* < 0.05; **, *p* < 0.01; ***, *p* < 0.001 in comparison to control group. #, *p* < 0.05 and ##, *p* < 0.01 in comparison to PAC group.

## Error in Figure

In the original publication [1], there was a mistake in Figure 1B as published. The figures in panel B (FA-Exo and FA-ExoPAC) were the same. The corrected Figure 1 appears below.

The authors apologize for any inconvenience caused and state that the scientific conclusions are unaffected. This correction was approved by the Academic Editor. The original publication has also been updated.

## Figures and Tables

**Figure 1:**
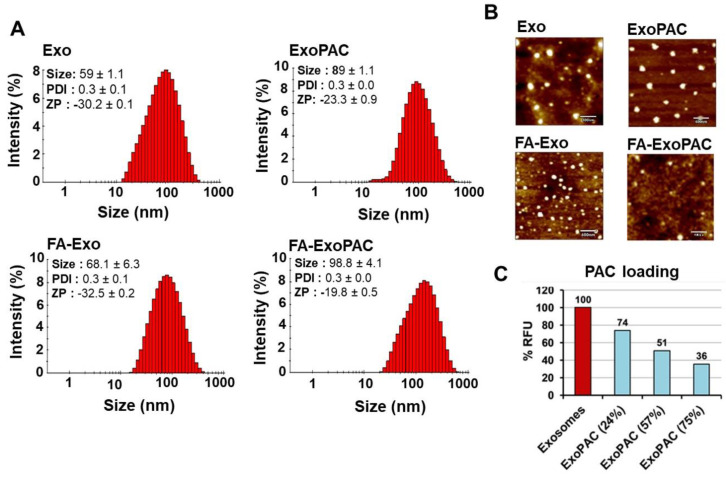
Characterization and drug loading of colostrum-derived exosomes. Size, polydispersity index (PDI), and zeta potential (ZP) of exosomes, FA-Exo, ExoPAC and FA-ExoPAC, analyzed by Zetasizer. Data represent mean ± SD from three preparations (**A**). Analysis of exosomes and ExoPAC by atomic force microscopy (AFM) after diluting with deionized water up to 10 μg/mL. For measurement, samples were placed on a silica wafer and air-dried for 30 min. AFM in tapping mode and aluminum-coated silicon probes were used for imaging (**B**). The bar diagram shows the quenching of autofluorescence from the exosomes following PAC loading (**C**). Higher quenching of fluorescence in the presence of higher drug load suggests a hydrophobic interaction of drug with exosomal proteins.
