# Supplementary material for: Targeted Oral Delivery of Paclitaxel Using Colostrum-Derived Exosomes
Source: Cancers (Basel). 2021 Jul 23;13(15):3700. doi: 10.3390/cancers13153700 (PMC8345039; doi:10.3390/cancers13153700)
Supplement: Supplementary file 1 [file cancers-13-03700-s001.zip › cancers-1284081-supplementary.pdf]

Supplementary Figures

Targeted Oral Delivery of Paclitaxel using Colostrum Derived Exosomes

Raghuram Kandimalla, Farrukh Aqil, Sara Alhakeem, Jeyaprakash Jeyabalan, Neha Tyagi, Ashish Agrawal, Jun Yan, Wendy Spencer, Subbarao Bondada and Ramesh C. Gupta

Supplementary Figure S1: Inhibition of A549 colony formation by PAC and ExoPAC.

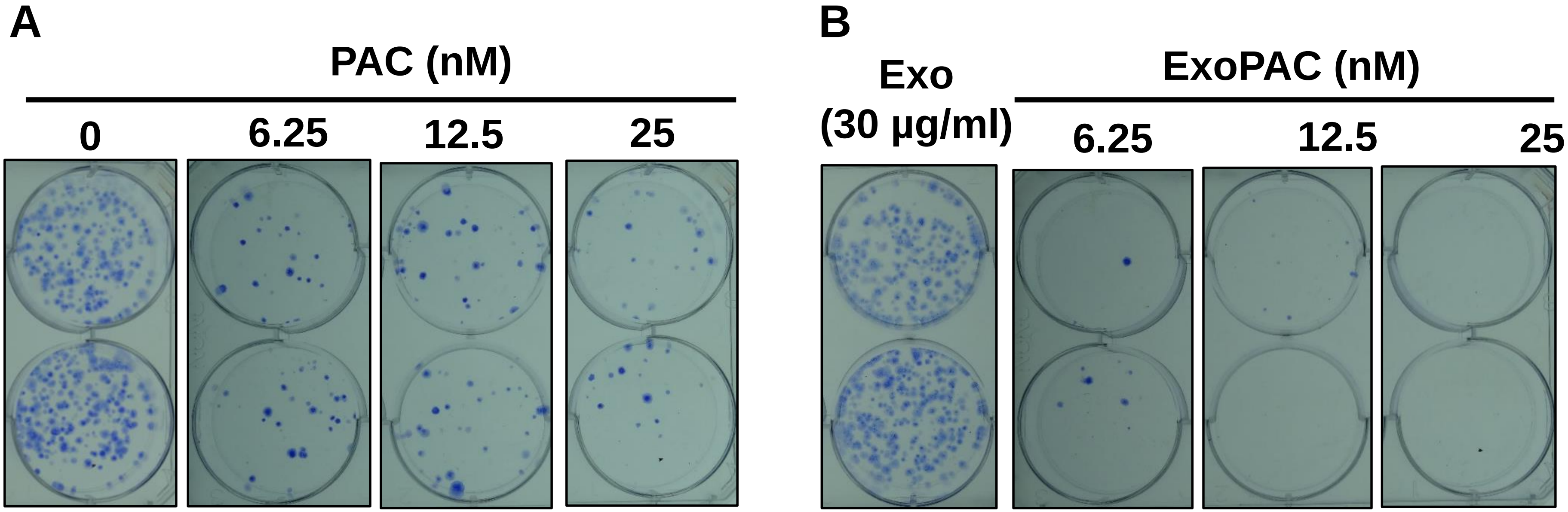

Representative images showing colony formation assay in drug-sensitive A549 cells. Lung cancer cells were seeded (500 cells/well) in a 6-well plate and incubated with different concentrations of PAC and ExoPAC. After 10 days, developed colonies were fixed, stained and counted manually. Exosome concentration was similar (30 µg/ml) in ExoPAC formulations.

Supplementary Figure S2: Inhibition of resistant A549TR colony formation by PAC and ExoPAC.

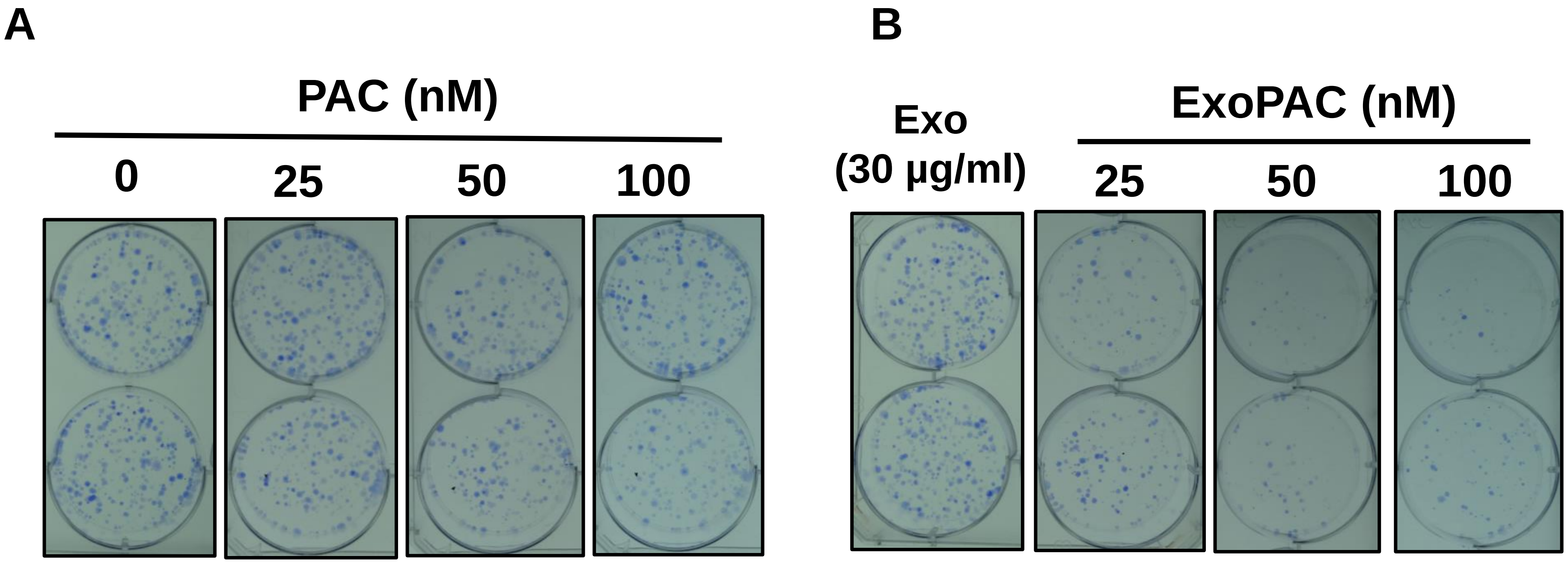

Representative images showing colony formation assay in drug-resistant A549TR cells. Lung cancer cells were seeded (500 cells/well) in a 6-well plate and incubated with different concentrations of PAC and ExoPAC. After 10 days, developed colonies were fixed, stained and counted manually. Exosome concentration was similar (30 µg/ml) in ExoPAC formulations

Supplementary Figure S3. Health index of the orthotopic lung tumor-bearing animals treated with PAC and ExoPAC.

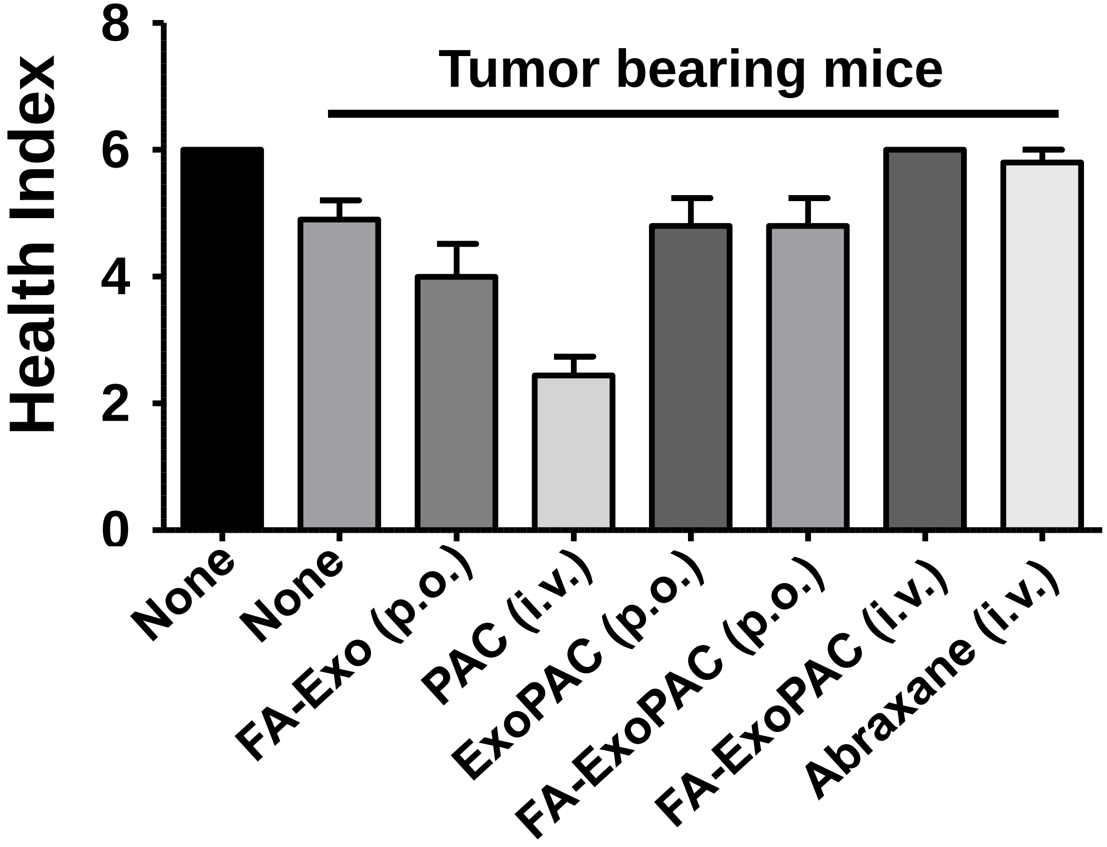

Figure shows the health index score (1-6; 6 being the best) of the animals in different treatment groups. Overall health index of the animals were assesses based on the locomotor activity, changes hair coat, diet consumption, weight loss, mortality during the study period and tumor severity in the thoracic cavity during surgery.
